# Task-shifting to nonexperts using artificial intelligence-guided point-of-care ultrasound: a cohort study of patient selection, image quality, and learning curves

**DOI:** 10.1093/ehjimp/qyag101

**Published:** 2026-07-06

**Authors:** Leah Wright, Cheng Hwee Soh, Bastian Seidel, Angus Baumann, Tony Mylius, Christopher Yu, Sudhir Wahi, Thomas H Marwick

**Affiliations:** Imaging Research Laboratory, Baker Heart and Diabetes Institute, Melbourne, Victoria 3004, Australia; Imaging Research Laboratory, Baker Heart and Diabetes Institute, Melbourne, Victoria 3004, Australia; Baker Department of Cardiometabolic Health, University of Melbourne, Melbourne, Victoria 3004, Australia; Ochre Medical Centre, Huonville, Tasmania, Australia; Alice Springs Hospital, The Gap, Northern Territory, Australia; Western Australian Country Health Service, Merredin District Hospital, Wheatbelt, Western Australia, Australia; Walgett Aboriginal Medical Service Limited, Walgett, New South Wales, Australia; Nepean Clinical School, University of Sydney, Sydney, New South Wales, Australia; Princess Alexandra Hospital, University of Queensland, Woolloongabba, Queensland, Australia; Imaging Research Laboratory, Baker Heart and Diabetes Institute, Melbourne, Victoria 3004, Australia; Baker Department of Cardiometabolic Health, University of Melbourne, Melbourne, Victoria 3004, Australia; Menzies Institute for Medical Research, Hobart, Tasmania, Australia

**Keywords:** Echocardiography, Hand-held ultrasound, Artificial intelligence, Nonexpert acquisition, Rural and remote

## Abstract

**Aims:**

To define rates of diagnostic image acquisition, clinical drivers of image quality and the learning curve for artificial intelligence (AI)-guided image acquisition on point-of-care ultrasound (AI-POCUS) in rural and remote communities.

**Methods and results:**

AI-guided image acquisition on point-of-care ultrasound was performed using AI software integrated with a desktop ultrasound system in 181 participants (65 ± 15 years, 47% female). A standardized training protocol included online material, lab attendance for 1 day, and online mentoring. Diagnostic-quality images were obtained from 72% of parasternal and 55% of apical images (*P* < 0.001). Scans were classified as ‘Diagnostic’ if diagnostic-quality images were obtained in the majority of parasternal and apical views (ACEP score ≥3) in ≥50% of windows for both apical and parasternal views. Body surface area (BSA) [OR 0.16 (0.05;0.50), *P* = 0.002] and hypertension [OR 0.50 (0.27;0.93), *P* = 0.03] were associated with diagnostic image quality in the apical window, whereas only hypertension [OR 0.43 (0.20;0.88), *P* = 0.024] was associated with diagnostic quality in the parasternal window. The learning curve was assessed by comparing the quality according to quantity of scans performed and professional background (nurse, health worker, or general physician). Physician-acquired scans [OR 3.85 (1.92;8.33), *P* < 0.001], scan 11th onwards [OR 2.86 (1.45;5.56), *P* = 0.002], and users who performed ≥20 scans [OR 3.58 (1.79;7.14), *P* < 0.001] predicted study completeness.

**Conclusion:**

In rural community practice, the learning curve associated with AI-POCUS diagnostic quality seems longer than reported in other studies from inpatient settings. In novice users, diagnostic quality is greater in the parasternal than the apical windows.

## Graphical Abstract

**Graphical Abstract:**
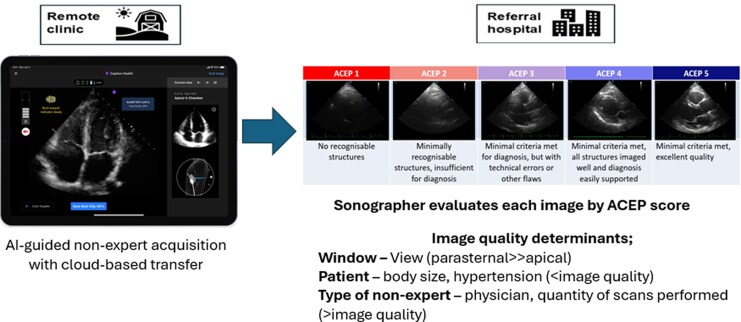

## Introduction

Access to transthoracic echocardiography (TTE) is a vital step towards correct diagnosis and appropriate management of heart failure (HF) and heart valve disease (HVD). Yet, access to TTE is unequal, with under-use in rural, remote, and disadvantaged areas.^1^ A critical contributing factor is the shortage of trained sonographers, driven by limited access to clinical training opportunities. Current sonographer workforce data show a national rate of 5.98 per 100 000 population in Australia, with regional variation to 3.65 per 100 000,^2^ similar to numbers in the USA.^3,4^

Artificial intelligence (AI) is increasingly integrated into clinical diagnostic pathways, with AI-guided point-of-care ultrasound (AI-POCUS) emerging as a novel tool at the intersection of AI and echocardiography. AI provides real-time guidance on probe positioning (e.g. sweeping, fanning, rotating) to optimize image acquisition^2^ and automated view recognition using neural networks trained on standard echocardiographic windows. Others have also shown the application of AI to guide image and disease quantification, including automated assessments of systolic function and potential future applications in valvular disease detection. These advances hold particular promise for regions where geographical and workforce challenges limit timely access to diagnostic services. In particular, delayed diagnosis of HVD,^5^ such as aortic stenosis (AS), remains a significant issue in older adults, with an estimation of over 100 000 individuals across Australia.^6^ Our prior community-based echocardiographic screening has revealed that many cases remained undiagnosed.^6^ This is in part attributable to limited access to echocardiographic assessment. AI-guided image acquisition on point-of-care ultrasound offers an opportunity to address this diagnostic bottleneck by task-shifting to nonexperts to acquire diagnostic-quality images with minimal training at the first care setting. Despite its promise, safe implementation of AI-POCUS requires a clear understanding of its current limitations, including the extent to which human oversight and training remain essential. Hence, this study sought to evaluate the effectiveness of AI-POCUS operated by nonexpert users for the recognition and management of undiagnosed disease. The primary objective was to identify the diagnostic quality of the obtained images. Additionally, we sought to determine whether patients’ or users’ characteristics (clinical background and number of scans performed) influence AI-POCUS image quality and the learning curve.

## Methods

### Data sharing

The data that support the findings of this study are available from the corresponding author upon reasonable request.

### Study design and setting

This study is a substudy to a randomized controlled trial (the AGILE-Echo trial, NCT05558605) and was aimed to demonstrate the feasibility and value of AI-POCUS in identifying patients with HVD or other cardiac dysfunction in rural and remote Australia,^7^ specifically in two rural towns with limited sonographer support and four remote communities without sonographers. This study followed the guidelines outlined in the Declaration of Helsinki and the National Statement on Ethical Conduct in Human Research. Written informed consent was provided by participants.

### Patient selection

Adult patients experiencing exercise intolerance in the presence of HF risk factors and people with suspected HVD (based on the presence of a murmur with or without symptoms or a history of rheumatic fever) were eligible for inclusion. Patients with diagnosed HF and HVD, taking cardioprotective treatment, with life-threatening comorbidity, or with inability to provide informed consent were excluded. In this study, we set out to report on 180 patients because this number exceeded the average size of comparable studies assessing the feasibility of AI-POCUS.

### Clinical assessment

Participants underwent a structured clinical assessment, during which sociodemographic data were collected. Functional capacity was evaluated using the Duke Activity Status Index.^8^ Additional clinical information included medical history, current medication use, and physical measurements. As these participants were at risk of HF, the 10-year risk of developing symptomatic HF, estimated using the Atherosclerotic Risk in Communities (ARIC-HF) risk score,^9^ was used to express risk level for the purpose of external validity.

### Selection and training of nonexperts

Each site nominated a nonexpert to perform the imaging, based on available time and interest. Of the 10 nonexpert users, including six nurses, two medical practitioners, and two Aboriginal health workers, none had previous exposure to training or performance of TTE.

Training was completed in two half-days (see Supplementary data online, *Table S1*). Participating sites were provided with online learning modules covering basic ultrasound physics, image acquisition techniques, and machine settings, as well as operator manuals for the echo machine and guidance software. One-day, in-person training sessions were provided at an echocardiography laboratory or on-site, focused on ‘hands-on’ location of echo windows, the importance of patient position, respiratory manoeuvers to optimize images, and feedback on acquired images. Ongoing feedback on image quality (at the request of the site) (including live guidance over video conferencing) was also provided when the nonexpert personnel returned to their sites.

### Imaging protocol

AI-guided image acquisition on point-of-care ultrasound was performed using the uSmart 3300 desktop ultrasound system (Terason, Burlington, MA) integrated with AI-guided software (Caption Health, Brisbane, CA). The echocardiographic views assessed included the parasternal long-axis (PLAX), parasternal short axis at the papillary muscle, mitral and aortic valve levels, and apical views—specifically the apical four-chamber (A4CH), apical long-axis (A3CH), and apical two-chamber (A2CH) views. Colour Doppler imaging could be used to assess valvular function. Echocardiographic measurements were performed by a single reader off-site (THM). The Teichholz method was used to calculate ejection fraction, and the ASE method was used for left ventricular (LV) mass, both from the PLAX.^10^

### Qualitative image analysis and learning-curve assessment

**Figure 1:**
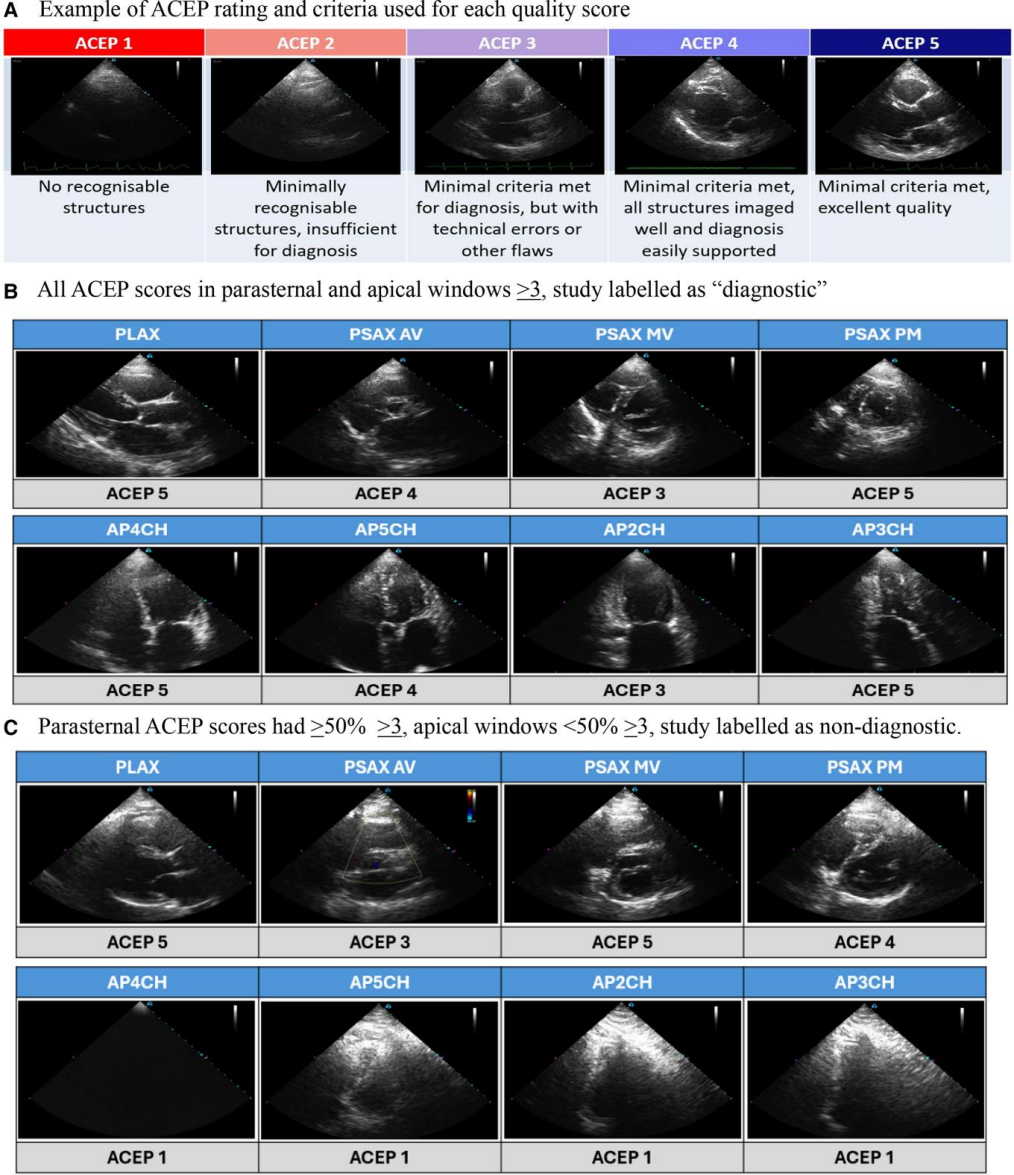
Categorization of image-quality AI-POCUS scans and example cases of diagnostic vs. non-diagnostic quality. (*A*) Example of ACEP rating and criteria used for each quality score. (*B*) All ACEP scores in parasternal and apical windows >3, study labelled as ‘diagnostic’. (*C*) Parasternal ACEP scores had ≥50% ≥ 3, apical windows <50% ≥ 3, study labelled as non-diagnostic.

All images were scored by a senior sonographer (LW) using the standardized five-point American College of Emergency Physicians (ACEP) scoring system for focused cardiac ultrasound.^11^ A second reader performed a blinded ACEP rating on 126 scans. A score of ≥3 was defined as meeting the minimum threshold for diagnostic adequacy (*Figure 1A*). Scans were also classified as ‘complete’ if diagnostic-quality images were obtained in the majority of parasternal and apical views (≥3 or 50% of images of sufficient quality in each window) (*Figure 1B* and *C*).

To assess the presence of a learning curve, three separate parameters were used. Diagnostic quality was compared between

- Users’ first 10 (1st–10th scans) against their subsequent scans (11th onwards).
- Users’ background (physicians against other allied health professionals).
- Users with ≥20 scans against users with <20 scans.

### Statistical analysis

Descriptive statistics were conducted for categorical, reported as number (percentage), and continuous variables, reported as mean ± standard deviation (if parametric) or median (interquartile range) (if nonparametric). Univariable logistic regression analyses were performed to identify factors associated with achieving a diagnostic-quality study, which was defined as obtaining diagnostic images in at least three standard echocardiographic views in both windows (parasternal and apical). Weighted kappa was used to compare interpretation by the primary study reader and the second reader. Multivariable logistic regression analyses adjusting for patients’ characteristics were also performed to identify the association between learning curves and diagnostic images.

To account for within-operator correlation arising from repeated scans acquired by the same health professional, a generalized linear mixed-effects model was computed with a logit link and a random operator intercept. Patient- and operator-level covariates [sex, body surface area (BSA), hypertension, scan experience, and physician status] were entered as fixed effects. This was strictly an exploratory analysis given a strong collinearity between scan experience and physician status [variance inflation factor (VIF) = 9.1]. We identified no missing data. Statistical analyses were conducted using SPSS (version 21) and R (R Foundation for Statistical Computing, version 4.4.2). A *P*-value of <0.05 was considered statistically significant.

## Results

### Patient characteristics

*Table 1* lists the characteristics of 181 participants. The predominant reason for inclusion was exercise intolerance (84%). Around a quarter of the participants were obese (25%), and more than half of them were either former (45%) or current smokers (17%). The mean ARIC-HF 10-year risk score was 1.3 ± 4.6%, and the DASI score of 38.7 ± 16.6 showed relatively good overall functional capacity. Echocardiographic measurements (*Table 2*) in images of adequate technical quality with an overall normal LV ejection fraction of 69 ± 12%. Abnormalities in imaging were found in 51 (28%) of participants, with the most frequent abnormalities being LA dilatation in 29 (16%) and LV hypertrophy in 19 (11%).

**Table 1:** Clinical characteristics

	Total participants (*n* = 181)
Age, years	65 ± 15
Female	85 (47%)
Body surface area	2.0 ± 0.3
Obese (≥30kg/m2)	46 (25.4%)
Smoking status	
Never smoked	67 (37.0%)
Ex-smoker	81 (44.8%)
Current smoker	30 (16.6%)
Aboriginal or Torres Strait Islander	32 (17.7%)
DASI	38.7 ± 16.6
Systolic blood pressure, mmHg	134 ± 18
Diastolic blood pressure, mmHg	78 ± 10
Heart rate, bpm	74 ± 14
Diabetes	31 (17.1%)
Hypertension	103 (56.9%)
Atrial fibrillation	16 (8.8%)
Angina	28 (15.5%)
ARIC-HF 10-year risk score, %	Median 1.3 (IQR 4.6)
Charlson Comorbidity Index	2.8 ± 2.2
Reasons for inclusion	
Exercise intolerance	132 (72.9%)
HVD concerns	28 (15.5%)
Both exercise intolerance and HVD	21 (11.6%)

ARIC-HF, Atherosclerotic Risk in Community Heart Failure risk score; DASI, Duke Activity Status Index; HVD, heart valve disease; LV, left ventricular.

**Table 2:** Echocardiographic parameters and abnormalities

Echo parameters (*n* = 125)	
LV IDD (cm)	4.8 ± 0.7
LVIDS (cm)	2.9 ± 0.7
LV PW (cm)	0.8 ± 0.1
IVSD (cm)	0.9 ± 0.1
LV mass indexed (g/m^2^)	76 ± 30
PLAX EF (%)	69 ± 12
Left atrial diameter	3.7 ± 0.5
Cardiac abnormalities	
Any abnormalities	51 (28%)
Left atrial dilatation	29 (16%)
Right atrial dilatation	10 (6%)
Right ventricular dilatation	13 (7%)
Mitral regurgitation	4 (2%)
Aortic regurgitation	4 (2%)
Concentric LVH	19 (11%)
Increased LV wall thickness	16 (9%)
Ejection fraction <55%	7 (4%)

IDD, Internal Diastolic Diameter; IDS, Internal Systolic Diameter; LV, left ventricular; PLAX EF (%), parasternal long-axis ejection fraction; PW, posterior wall.

AI-guided image acquisition on point-of-care ultrasound was completed by 10 (two physicians and eight allied health professionals) nonexperts. There was a considerable variation in the time taken to perform the scans, with a median of 17 min (IQR 32). For user characteristics, 110 scans were performed by a primary care physician (61%), 107 (59.1%) scans were completed by users on their 11th scans onwards, and 96 (53%) scans were done by users with ≥20 scans.

### Image quality

**Figure 2:**
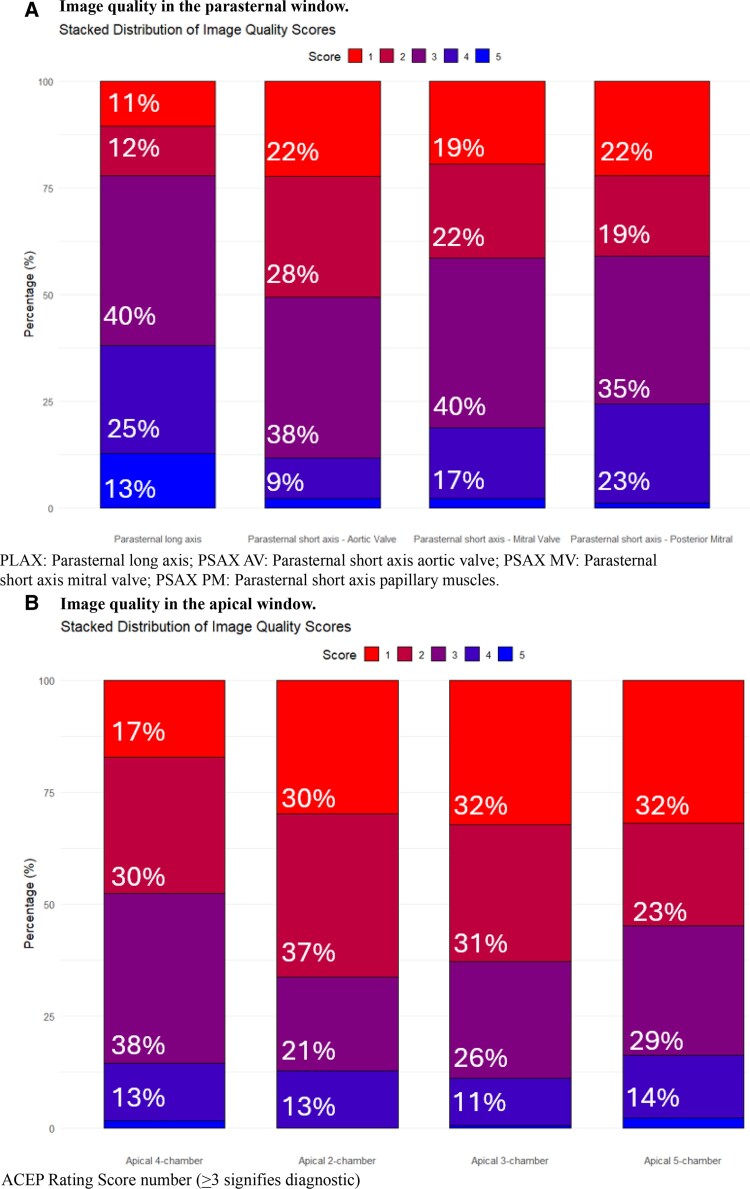
(*A*) Image quality in the parasternal window. PLAX, parasternal long-axis; PSAX AV, parasternal short-axis aortic valve; PSAX MV, parasternal short-axis mitral valve; PSAX PM, parasternal short-axis papillary muscles. (*B*) Image quality in the apical window. ACEP Rating Score number (≥3 signifies diagnostic).

A total of 131 participants had diagnostic-quality parasternal windows (72.4%), while 99 participants had diagnostic-quality apical windows (54.7%). *Figures 1* and *2* show the image quality in each view using the standard criteria from the ACEP score, and the parasternal long-axis showed the greatest proportion of diagnostic images (ACEP ≥3). The apical windows showed varying degrees of quality, although in no view did the proportion of scans with ACEP score ≥3 exceed 80%. The weighted kappa between was moderate for inter-reader variability (see Supplementary data online, *Table S2*).

### Clinical associations of image quality

Univariable logistic regression showed that patients’ BSA [OR 0.23 (0.07–0.70), *P* = 0.012] and hypertension [OR 0.32 (0.17–0.60), *P* < 0.001] were significantly associated with complete diagnostic scans (diagnostic at both parasternal and apical windows), while sex [male OR 1.41 (0.78–2.57), *P* = 0.256] and age [OR 0.98 (0.96–1.00), *P* = 0.091] were not. Specifically in the apical window, BSA and hypertension were associated with image quality (*Table 3*), while only hypertension remained a significant factor in obtaining diagnostic image in parasternal window.

**Table 3:** Univariate associations of study quality

	Parasternal windows diagnostic, *n* = 131		Apical windows diagnostic, *n* = 99		Overall complete study, *n* = 81	
	OR (95% CI)	*P*	OR (95% CI)	*P*	OR (95% CI)	*P*
Male	1.79 (0.92–3.50)	0.087	0.85 (0.47–1.53)	0.579	1.41 (0.78–2.57)	0.256
Age (years)	0.98 (0.95–1.00)	0.082	0.99 (0.97–1.01)	0.169	0.98 (0.96–1.00)	0.091
BSA (m^2^)	0.77 (0.23–2.63)	0.678	0.16 (0.05–0.50)	0.002	0.23 (0.07–0.70)	0.012
Hypertension	0.43 (0.20–0.88)	0.024	0.50 (0.27–0.93)	0.03	0.32 (0.17–0.60)	<0.001

BSA, Body surface area.

### Learning curve and image quality

Multivariable regression analyses showed that physician-acquired scans [OR 3.85 (1.92–8.33), *P* < 0.001], scan 11th onwards [OR 2.86 (1.45–5.56), *P* = 0.002], and users who performed ≥20 scans [OR 3.58 (1.79–7.14), *P* < 0.001] were significant predictors of study completeness after adjusting for sex, BSA, and hypertension (*Table 4*).

**Table 4:** Multivariable logistic regression between overall study qualities with learning-curve markers

Effect of physician scanning on study quality			Effect of moderate scan experience on study quality			Effect of > moderate scan experience on study quality		
	OR (95% CI)	*P*-value		OR (95% CI)	*P*-value		OR (95% CI)	*P*-value
Physician scanned	3.85 (1.92–8.33)	<0.001	Scan 11th onwards	2.86 (1.45–5.56)	0.002	Users with ≥20 scans	3.58 (1.79–7.14)	<0.001
Male	2.35 (1.11–5.17)	0.028	Male	2.35 (1.13–5.09)	0.025	Male	2.41 (1.14–5.31)	0.024
BSA	0.13 (0.03–0.55)	0.008	BSA	0.16 (0.04–0.66)	0.013	BSA	0.12 (0.03–0.52)	0.006
Hypertension	0.28 (0.14–0.56)	<0.001	Hypertension	0.33 (0.17–0.64)	0.001	Hypertension	0.32 (0.16–0.63)	0.001

BSA, Body surface area.

In mixed-effects logistic regression accounting for clustering by operator (*Table 5*), higher BSA (OR 0.10, 95% CI 0.02–0.44) and hypertension (OR 0.29, 95% CI 0.14–0.58) were associated with lower odds of overall diagnostic image quality, while sex was associated with higher odds (OR 2.72, 95% CI 1.26–5.84). Scan count (log10) (OR 0.71, 95% CI 0.48–1.06) and physician status (OR 0.47, 95% CI 0.10–2.21) were not independently associated with diagnostic quality.

**Table 5:** Mixed-effects logistic regression (random operator intercept) for factors associated with overall diagnostic image quality

Overall complete study		
	OR (95% CI)	*P*-value
Male	2.72 (1.26–5.84)	0.013
BSA	0.10 (0.02–0.44)	0.003
Hypertension	0.29 (0.14–0.58)	0.001
Log(Scan count)	0.71 (0.48–1.06)	0.101
Physician scanned	0.47 (0.10–2.21)	0.391

BSA, Body surface area.

## Discussion

In this study, AI-POCUS was found to be feasible in rural community practice, although the current success rate would be considered more suitable for *ad hoc* use when sonographers are unavailable, rather than task-shifting on a routine basis. Also, AI-POCUS would seem better fitted to a triage style use, where normal studies could be excluded, but those with evidence of systolic dysfunction or evidence of valvular heart disease would proceed to a full study (especially as AI systems do not currently guide colour or spectral Doppler). ‘Super users’ may be an appropriate strategy at sites where this is considered for clinical use, as a learning-curve effect is observed. AI-guided image acquisition on point-of-care ultrasound performed by nonexpert users was most effective for acquiring diagnostic-quality images (in nearly 80% of patients) in the parasternal windows. In contrast, image quality in the apical views was more variable, with only about half of the participants’ windows meeting the diagnostic threshold.

Importantly, increasing the number of scans performed led to improvements in diagnostic image quality, with the clinical background of those acquiring images also being a factor.

### Clinical relevance

The reason that these results are important is that there is variation of access to TTE, based on location and access to cardiology specialists.^1^ People living in rural and remote Australia (including but not restricted to the Aboriginal community) have the lowest access to cardiac imaging.^1^ Rural areas struggle with health professional shortages, distance to services, and inadequate service ability with this often compounded by socioeconomic disadvantages.^12^ The Australian Commission on Safety and Quality in Health Care reports that rates of standard echocardiography are significantly lower in outer regional and remote areas compared to major cities and inner regional regions, indicating less frequent availability of echocardiography services.^13^ These factors, combined with the devastating consequences of RHD in Indigenous communities,^14^ highlight how the results of this study, showing feasibility of AI-TTE by nonexperts, could be of benefit to rural cardiovascular disease outcomes. While provision of the appropriate equipment is essential, training and education will be vital to the uptake and success of AI-enhanced acquisition.

### Testing of AI-guided TTE

*Table 6* summarizes currently published studies in which AI-POCUS has been applied in a clinical population.^2,3,15–19^ Although marketed as a tool for novices, most studies have incorporated a training programme before use. This has varied—some papers comment on a 2-week training period,^20^ although short periods of 1 or less day are most common.^15,17^ In our experience, differences in clinical background (physician vs. other health worker) did appear to influence learning curves, confirming the findings of other studies.^3^ Both the sites and users self-selected, based on available time and interest. While this selection process may appear to provide a ‘best-case scenario’ for clinical application of AI imaging, it likely reflects the selection process in adoption of technology.

**Table 6:** Review of current AI-POCUS literature and image quality, sample size, and learning-curve characteristics

Study (author, year)	Sample size and population	Equipment and protocol	Personnel and training	Quality measures	Key results/quality levels
Narang et al., 2021^2^	240 patients In-/outpatients Exclusions: unable to lie flat, severe chest deformity, consent issues BMI stratified evenly	Caption Guidance (Caption Health) Imaging Protocols: 10 standard views: PLAX PSAX AV PSAX MV PSAX PM AP4CH AP5CH AP2CH SC 4CH SC IVC	8 nurses 1-h didactic 9 practice scans	Qualitative assessment: LV/RV size & function Pericardial effusion Valve structure IVC size Clinical interpretability: ≥3/5 expert agreement	Sufficient quality for: LV size: 95.7% LV function: 96.6% RV size: 92.5% RV function: 92.9% Pericardial effusion: 99.6% Aortic: 90.6% Mitral: 93.3% Tricuspid: 95.2%
Cheema et al., 2021^15^	Case study series of 5 by critical care staff with no formal echocardiography training	Caption Health	Series from point-of-care ultrasound	Real-world use of technology in the COVID ICU	Bedside use can affect decision-making and patient care
Schneider et al., 2021^16^	19 novices scanned 3 patients each	Caption Health Imaging protocol PLAX Ap4CH Ap 2CH	19 first-year medical students 2.5-hour online training 10 students also received 2 h of hands-on training	ACEP scores are generated via a validated prediction algorithm.	Diagnostic-quality images were obtained in PLAX 33/57 (58%) Apical 4CH 49/57 (86%) Apical 2CH 39/57 (68%) 25/57 (44%) of scans had diagnostic quality in all three images Excellent agreement in EF in those with adequate images
Peck et al., 2023^17^	50 patients 25 RHD, 25 normal	Caption: Imaging Protocol 7 standard transthoracic echocardiographic views: PLAX PLAX colour mitral PLAX colour aortic valve AP4 AP4 colour mitral AP5 AP5 colour aortic valve.	36 novices Each scanned 8–10 participants 7-view protocol <5h training (didactic + hands-on with AI guidance) Questionnaire post-training	Diagnostic interpretation (RHD) ACEP scores	Diagnostic interpretation >90% for RHD AR, 79%; AS, 50% ACEP (mean %>3) PLAX, 3.5 (81%) AP4CH: 3.2 (74%) AP5CH: 2.4 (38%) No AP2/SC images
Mor-Avi et al., 2024^3^	240 patients 5 excluded for poor acoustic windows Typical tertiary care echo population 65.5% never consumed alcohol, 67.4% never smoked	UltraSight v1.1.0 (Lumify, Philips) Imaging Protocol: 10 standard views: PLAX PSAX AV PSAX MV PSAX PM AP4CH AP5CH AP2CH SC 4CH SC IVC	6 nurses, 3 residents 8-h lectures (cardiac anatomy, echo basics) 8 practice scans per participant	5 readers ACEP score Qualitative & diagnostic interpretation Consensus among readers	ACEP >3: PLAX: 86% (79–93%) AP4: 94% (90–97%) AP2: 85% (78–90%) AP3: 83% (76–91%) SC-IVC: 68% (61–75%) No association with clinical markers and image quality
Mears et al., 2024^18^	184 scans Neurology ICU	Caption Guidance AI v1.1.1: Imaging Protocol: PLAX PSAX (? No comment on level), AP4CH AP2CH SC4CH SC IVC	42 operators (median 2 scans each) Training Sessions: 10-min intro lesson (device use, hands-on, survey) Time for questions	3 readers ACEP score Automated EF change of management Operator confidence	% Interpretable: SC4CH: 52.6% PSLAX: 50.6% AP2CH: 28.4% Pathology diagnosis: LV: 63.4% RV: 52.6% IVC: 34.8% Pericardial effusion: 47.2% Learning curve: lower experience, better images but longer scan time Clinical impact: 37.3% of scans changed management (fluid management most common)
Papadopoulou et al., 2024^19^	115 patients’ oncology department Exclusions: breast implants, thoracic surgery, structural deformities	Kosmos HUD (EchoNous) Imaging Protocol: AP4CH AP2CH	5 doctors, 3 nurses Training session Theoretical (2 × 2h lectures) Hands-on (2h, twice/week, 1 month)	Only AP4 and AP2 views Max 8 min/scan Image quality sufficient in 111/115 HUD acquisition: good, 21%; moderate, 54%; poor, 25%	AI-EF feasible: Cardiologist: 96% Senior oncologist: 94% Junior oncologist: 93% Oncology nurse: 89% Learning curve: significant improvement in 2 users (measured r values and compared)
Huang et al., 2024^20^	100 symptomatic patients with suspected HF	EchoNous Kosmos hand-held echo, with AI-automated reporting by Us2.ai (AI-enhanced novice pathway)	1 study coordinator: Theoretical Hands-on 2 weeks (first week shadowing sonographer, second week image acquisition)	AI-measurable EF Accuracy of AI-enhanced echo to detect EF<50% Learning curve	96 (96%) of studies had sufficient quality for use with AI-EF programme (Us2.ai) AUC for detecting EF<50% was 0.88 Learning rate plateaued between scans: 40–60

The overall success rates of diagnostic-quality images vary greatly depending on the study. The literature does show apparent differences between image quality and the amount of training performed, with those with the shortest training protocols displaying the lowest percentage of interpretable images.^18^ The operator background has previously shown to be a factor in image quality,^19^ possibly due to the previous exposure to TTE that occurs with medical training. For AI-POCUS to fulfil its promise of improving access to TTE, there may be merit in users engaging in a structured training programme. Participant clinical factors show mixed results—this is likely due to the heterogeneity of background in the clinical studies, with some stratifying based on BMI.^2^ Our clinical cohort was selected on the basis of nonspecific symptoms, with BMI, smoking status, hypertension, Indigenous background, and age being the most prominent risk factors for cardiac disease.

### Quality and diagnostic capability

Our rates of diagnostic-quality images are considerably less than previous studies comparing diagnostic rates of AI-POCUS technology with clinical sonographers, which found near-perfect associations.^3^ There are several potential explanations. First, our study may reflect the challenges of training outside of a cardiology department, among staff who do not have general exposure to cardiac imaging techniques, nor the mentoring from sonographers and specialists that might be available in a hospital environment. These considerations are supported by similar results in a previous community-based cohort in which novice providers provided diagnostic-quality studies, but with higher image-quality scores in the parasternal long-axis than the apical four-chamber.^17^ Second, participants in our study accurately represent an undifferentiated community cohort, among whom image quality was unknown. This is likely representative of how AI-POCUS might perform in the ‘real world’ of community practice.

One of the situations in which AI-POCUS hold promise is in screening for rheumatic heart disease (RHD). The Single Parasternal Long-Axis Sweep (SPLASH) technique has been shown to improve the efficiency of RHD screening.^21^ At present, this approach is based on training nurses and community health workers, but the shortcoming is that expertise is lost when these staff rotate away to another location. As the results of our current study show that AI-POCUS is most feasible for PLAX imaging, AI might be used to shorten the SPLASH learning curve of nonexperts. Current AI algorithms would not fit the requirements, due to the dynamic and ‘sweeping’ nature of the SPLASH protocol. If a training model were produced, the role of AI-POCUS for the detection of mitral valve disease seems promising.^17^

#### Image-quality analysis

We used the ACEP score to quantify image quality. Overall, other papers have found a ‘moderate’ agreement using ICC.^22^ The relatively modest kappa values likely reflect the inherent subjectivity of image-quality assessment, with variability arising from differences in reader experience, familiarity with grading systems, and individual thresholds for acceptability. This is further compounded in lower-quality studies, where reduced reproducibility and increased interobserver variability are expected. Although prior studies assessing agreement in ACEP scoring have used intraclass correlation coefficients (ICC), with our data demonstrating values above 0.8, weighted kappa was selected following statistical consultation as the more methodologically appropriate approach. While automated image analysis may represent an ideal future method to reduce human bias,^23^ the present findings highlight the challenges of achieving high interobserver agreement in qualitative image assessment, particularly in technically difficult datasets.

#### Limitations

This study has a number of limitations. First, it was conducted in a few clinical sites with a handful of clinicians, with a wide range in number of echo studies done. It may not be generalizable to other health systems where the target trainees may have different experience or clinical background. Second, the study used one particular AI–echo platform, and other platforms may have a different performance. Third, assessment of quality was limited to greyscale ultrasound, but clearly the use of colour Doppler will be important in the clinical application of this modality. Fourth, the study population had suspected rather than overt disease, and diagnostic performance may be different based on the distribution of heart disease. Fifth, this educational intervention was designed to be completed before the involved remote practitioners started work and additional feedback from online support was not uniform or quantified. Subsequent work is planned to better measure quality control during and after training. Ongoing support was not standardized, which risks bias as it is purely informed by users, and not based on a quality assessment.

Although the interpretation of single views has been useful for some indications, a complete study for most indications requires multiple views. In this sense, high scores for image quality in individual views may be optimistic in many clinical settings. These findings have important implications for understanding patient selection and criteria for triaging to a standard TTE. Ejection fraction and LA size can be estimated from a single PLAX view, but if there is a clinical suspicion of CAD, not LV myocardial segments will likely be visualized adequately.

This study sought the efficacy of task-shifting in symptomatic people without diagnosed disease—the results could be different in people with known HF or HVD. Finally, these observations are applicable in rural and remote Australia, but their generalizability to other settings is unproven.

#### Conclusions

AI-guided image acquisition on point-of-care ultrasound appears feasible in rural community practice and may assist with solving blocks to patient access. However, the learning curve associated with diagnostic quality seems longer than previously reported in studies which took place in an inpatient setting. In novice users, diagnostic quality is greater in the parasternal than the apical windows, highlighting the potential for revisiting quantitation in this view.

## Clinical perspective

The dependence of TTE on expert acquisition contributes to problems with TTE access. The development of AI-POCUS means that nonexpert users (nurse, health worker, or general physician) can be guided to acquire TTE images. However, there are limited studies on the diagnostic yield of AI-POCUS in the community—which is where it is most needed. In this study of 181 participants (65 ± 15 years, 47% female) recruited from two small rural towns and four remote communities across Australia, we sought to define rates of diagnostic image acquisition, clinical drivers of image quality, and the learning curve for AI-POCUS acquisition at rural sites. A standardized training protocol included online material, lab attendance for 1 day, and online mentoring. Diagnostic-quality images were obtained from 78% of parasternal and 52% of apical images (*P* < 0.001). In the apical window, BSA [OR 0.16 (0.05;0.50), *P* = 0.002] and hypertension [OR 0.50 (0.27;0.93), *P* = 0.03] were associated with reduced quality studies in the apical window, whereas only hypertension [OR 0.43 (0.20;0.88), *P* = 0.024] was associated with diagnostic quality in the parasternal window. Physician-acquired scans [OR 3.85 (1.92;8.33), *P* < 0.001], scan 11th onwards [OR 2.86 (1.45;5.56), *P* = 0.002], and users who performed ≥20 scans [OR 3.58 (1.79;7.14), *P* < 0.001] were significant predictors of study completeness. In rural community practice, the learning curve associated with AI-POCUS diagnostic quality seems longer than reported in hospital practice. In novice users, diagnostic quality is greater in the parasternal than the apical windows, offering the potential for quantitation in this view.

## Supplementary Material

qyag101_Supplementary_Data

## Data Availability

The data used to support the findings of this study are included within the article. Further data are available on request from the corresponding author.

## Lead author biography

Tom Marwick is a clinician–scientist from Australia. After obtaining his medical degree from the University of Melbourne, he completed certification in Medicine and Cardiology and undertook international training (Imaging Fellowship, Cleveland Clinic; PhD at Universite Catholique de Louvain, Brussels; MPH at Harvard). He has had extensive international research and clinical experience, with ongoing international collaborations. Marwick is a Professor of Medicine at the Universities of Melbourne and Tasmania. He is a practising cardiologist/cardio-oncologist at the Royal Hobart Hospital. Marwick’s career-long interest has been in myocardial imaging—mainly in cardio-oncology and HF.
