# Supplementary material for: Task-shifting to nonexperts using artificial intelligence-guided point-of-care ultrasound: a cohort study of patient selection, image quality, and learning curves
Source: Eur Heart J Imaging Methods Pract. 2026 Jul 6;4(3):qyag101. doi: 10.1093/ehjimp/qyag101 (PMC13335798; doi:10.1093/ehjimp/qyag101)
Supplement: qyag101_Supplementary_Data [file qyag101_supplementary_data.docx]

**Appendix.**

**Supplementary Table 1. Training protocol**

| 1^st^ half-day (or equivalent in trainee’s time) - Online | 1. Information on the trial 2. Information on principles of ultrasound, equipment setting and getting the right views   <https://www.youtube.com/watch?v=9LjtPWfsjFw> Principles of ultrasound  <https://www.youtube.com/watch?v=m9s5AD_lBkY> Obtaining views  <https://www.youtube.com/watch?v=Cg8Mzbc4TBY> Performing a full study  <https://www.youtube.com/watch?v=4Yu-ftQzHZM> equipment settings   1. Information of the Caption Health machine – from manuals and sites;   <https://www.caption-care.com/technology>  [Terason - uSmart 3200T Community, Manuals and Specifications \| MedWrench](https://www.medwrench.com/equipment/6964/terason-usmart-3200t) |
| --- | --- |
| 2^nd^ half-day – Practical training in the echo lab | The goal of this session was to witness and mimic study acquisitions I the presence of a sonographer, including positioning the patient, the importance of respiratory manoeuvres and the "buttonology" of the machine. |
| Online support | Support provided though video-conferencing to trouble-shoot during acquisitions at the remote site. There was no standard “dose” and the focus was on trouble-shooting and feedback, which was given to all clinicians by authors LW and TM. In general, this resource was provided for the first 5-10 studies, but it was at the request of the non-expert clinicians, and subject to their and the trainers’ availability. There was no re-training (the sites are several hundred kilometers from the training echo lab and thousands of kilometers from the investigators. |

**Supplementary Table 2: Agreement between two readers for ACEP scores**

| **Echocardiographic window** | **Weighted Kappa** |
| --- | --- |
| PLAddX | 0.314 |
| PLAX AV | 0.482 |
| PLAX PM | 0.365 |
| PLAX MV | 0.327 |
| AP4CH | 0.319 |
| AP5ch | 0.399 |
| Ap2ch | 0.459 |
| AP3CH | 0.514 |
| PLAX Diagnostic | 0.421 |
| Ap4ch Diagnostic | 0.399 |

PLAX; Parasternal Long Axis, AV; Aortic Valve , PM; Papillary Muscle, AP4CH; Apical four chamber, AP5CH; Apical five chamber, AP2CH; Apical two-chamber, AP3CH; Apical long-axis;
